# Diagnostic Confidence of Run-Off CT-Angiography as the Primary Diagnostic Imaging Modality in Patients Presenting with Acute or Chronic Peripheral Arterial Disease

**DOI:** 10.1371/journal.pone.0119900

**Published:** 2015-04-02

**Authors:** Thomas Werncke, Kristina Imeen Ringe, Christian von Falck, Martin Kruschewski, Frank Wacker, Bernhard Christian Meyer

**Affiliations:** 1 Klinik für Radiologie, Charité Universitätsmedizin Berlin, Berlin, Germany; 2 Institute of Diagnostic and Interventional Radiology, Hannover Medical School, Hannover, Germany; Shenzhen institutes of advanced technology, CHINA

## Abstract

**Objectives:**

To investigate the reliability of CT-angiography of the lower extremities (run-off CTA) to derive a treatment decision in patients with acute and chronic peripheral artery disease (PAD).

**Materials and Methods:**

314 patients referred for run-off CTA were includ-ed in this retrospective study. First, diagnostic confidence of run-off CTA to derive a treat-ment decision was assessed in an interdisciplinary vascular conference using a 2 point scale (sufficient or not sufficient diagnostic confidence) and compared with the image quality eval-uated by two readers in consensus in four different levels (abdominopelvic, thigh, calf, foot arteries). Second, reliability of treatment decision was verified in all patients undergoing re-vascularization therapy.

**Results:**

Diagnostic confidence of run-off CTA to derive a treatment deci-sion was sufficient in all patients with acute and in 97% of patients (215/221) with chronic PAD, whereas the rate of run-off CTA with non-diagnostic image quality was considerably higher in the calf and foot level (acute vs. chronic; calf: 28% vs.17%; foot: 52% vs. 20%). Reliability of treatment decision was superior for patients with chronic (123/133 = 92%) than for patients with acute PAD (64/78 = 82%, P = 0.02).

**Conclusion:**

Run-off CTA is a reliable imaging modality for primary diag-nostic work-up of patients with acute and chronic PAD.

## Introduction

Peripheral arterial occlusive disease (PAD) is a common cardiovascular disorder in elderly patients with a reported prevalence of more than 6% in patients older than 65 years [1]. If claudication symptoms progress and limit the quality of life despite appropriate conservative treatment or if the patient suffers from acute limb ischemia, assessment of the whole arterial tree is recommended according to the Transatlantic Inter-Society Consensus conference for the Management of Peripheral Arterial Disease [1]. The overall objective of imaging and revascularisation therapy is to relieve pain, improve the function of the legs and thereby the quality of life [1].

Although digital subtraction angiography (DSA) is still the gold standard for the visualization of lower limb arteries, non-invasive imaging modalities such as CT-angiography (CTA) and MR-angiography (MRA) are increasingly recommended and used for clinical work-up of patients with advanced PAD. In recent meta-analyses, MRA and CTA of the lower extremities (run-off MRA and run-off CTA) showed an equal pooled mean sensitivity and specificity (MRA: 93–95% and 94–96%, respectively; CTA 95–96% and 95–96%, respectively) for the detection of haemodynamic relevant stenoses >50% and total occlusions [2–4]. However, these pooled studies as well as most of the studies evaluating CTA and MRA in patients with PAD, report the diagnostic accuracy on a per–lesion basis and not on a per-patient basis [5,6] and therefore do not take into account the sequence of stenosis if multiple stenoses are present.

In contrast to DSA and MRA, in which a luminogram is created based on subtraction techniques, the primary run-off CTA dataset has to be post-processed using threshold-based region growing bone removal techniques. As calcified plaques remain in the processed dataset, they can obscure the lumen margins by blooming effects and get visualized in maximum intensity projection (MIP) images used for reading and communication of the findings. Therefore, severe vessel calcifications can interfere with the delineation of the lumen and therefore impair stenosis quantification [7]. On the other hand, visualization of calcified lesions may be helpful to either decide for endovascular or surgical treatment of the patient.

There is only sparse literature [2,8–10] investigating the clinical value and patient outcome if run-off CTA is used as primary imaging modality in patients with chronic PAD. To the best of our knowledge, there is no published study evaluating the clinical value of run-off CTA in patients with acute PAD for a larger patient collective.

The purpose of this retrospective study was to investigate the reliability of run-off CTA to derive a treatment decision in patients with acute and chronic peripheral artery disease.

## Materials and Methods

This retrospective study was institutional review board (EA4/116/11, local ethics committee, Charité Universitätsmedizin Berlin) approved with a waiver of consent granted. Patient records and information were anonymized and de-identified prior to analysis.

### Patient population

**Fig 1:**
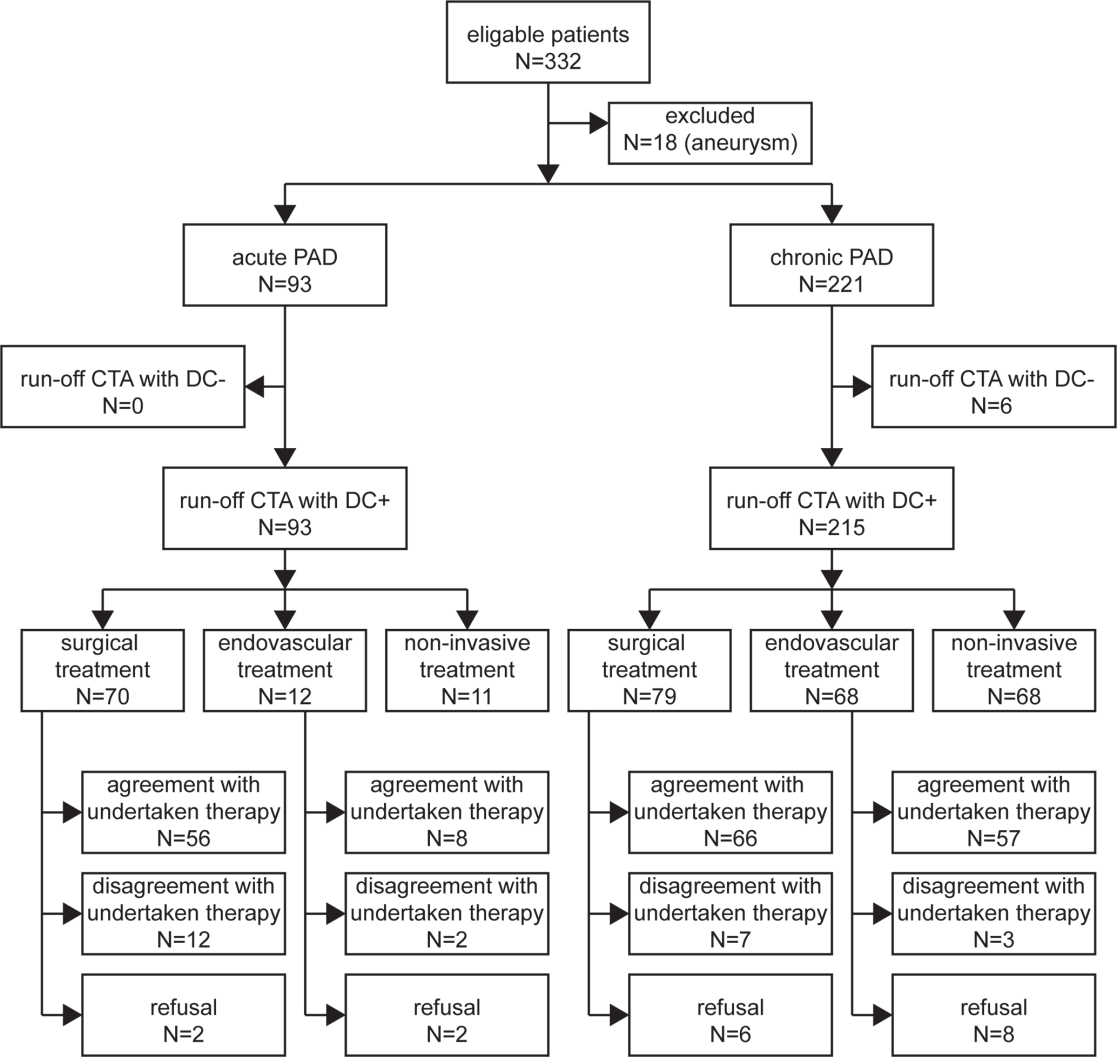
Flow diagram of patients with acute and chronic PAD. DC+/DC−: sufficient / insufficient diagnostic confident run-off CTA.

A total of 332 consecutive patients referred from 01/2008–08/2011 for run-off CTA because of clinical symptoms of acute or chronic PAD were screened for study inclusion (Fig. 1).

Patients were included in this study if the institutional, standardized run-off CTA protocol was completed and available. This protocol is completed by the supervising radiologist in all patients referred for run-off CTA in order to obtain and document all relevant clinical information. This includes demographic data, patient history, clinical symptoms of PAD, Fontaine stage, presence of cardiovascular risk factors (arterial hypertension, hyperlipidaemia, history of nicotine abuse, diabetes mellitus, renal impairment defined as serum creatinine level >1.4 mg/dl and chronic haemodialysis). Main purpose of this document is to provide essential information to the reporting vascular and interventional radiologist. Secondly, this protocol is used to document the quality of run-off CTA as a part of quality assurance in our department as well as the intended therapy based on it.

Exclusion criteria were as follows: patients with combined diabetes mellitus and a history of chronic haemodialysis longer than five years (according to our institutional standard operating procedures these patients are directly referred for DSA because severe vessel wall calcifications must be expected [7]); abdominal aortic aneurysm (defined as aortic diameter > 30 mm) or iliac artery aneurysm (defined as vessel diameter > 20 mm), assuming a changed haemodynamic situation [11]. Thus, 314 patients (204 males, mean age 67 ± 9 years; 110 females, mean age 73 ± 12 years) were included in this study.

Evaluation of clinical symptoms was based on the Fontaine classification [1] combined with the classification of limb ischemia proposed by Callum et al. [1,12]. PAD was classified as acute, if clinical symptoms lasted less than 14 days, or if symptoms and signs worsened within this time period. If clinical symptoms were stable for more than 14 days, PAD was defined as chronic.

### Image acquisition

Run-off CTA (covering the range from the costodiaphragmatic recess to the forefoot) was conducted on a 64-slice MDCT system (Somatom Definition, Siemens Healthcare, Forchheim, Germany) using following acquisition parameters: tube voltage 120 kVp, reference tube current-time product 120 mAs with tube current modulation CareDose4D, rotation time 0.33 s; collimation 2 x 32 x 0.6 mm, pitch 0.75, field of view 330 mm. Prior to imaging, tourniquets were tied round each thigh in order to reduce venous return and improve image quality [13]. 100 ml of Iomeprol 400 mg I/ml (Imeron 400, Bracco, Milan, Italy) followed by a 60 ml saline flush at a flow rate of 4.0 ml/s were injected in all patients using a dual barrel injector (Stellant, Medrad, Volkach, Germany) via a 20 G or larger intravenous cannula located in an antecubital vein. Arterial phase images were obtained 3 s after bolus detection in the suprarenal aorta (threshold 250 HU). Images were reconstructed using a soft kernel (B25f) with an effective slice thickness of 1.0 mm (reconstruction interval 0.7 mm).

### Post-processing and image analysis

Post-processing and image analysis were conducted on a Thin Client PACS System (VisageCS 7.0, Visage Imaging GmbH, Berlin, Germany). A semi-automatic bone removal algorithm was applied and rotating maximum intensity projections (MIP; viewing range of 180° at 15° intervals) of three overlapping regions were created as follows: Region 1: suprarenal aorta—femoral bifurcation; region 2: common femoral artery—origin of the tibiofibular trunk; region 3: P3 segment of the popliteal artery—forefoot arteries). If vessel calcifications interfered with interpretation of the MIP images, additional curved planar reformations (CPR) were generated.

To investigate the reliability of run-off CTA to derive a treatment decision, the diagnostic confidence (DC), the image quality (IQ) and the reliability of the treatment decision in patients receiving a revascularization therapy was assessed.

### Diagnostic confidence

In our hospital, all run-off CTA studies are presented daily in an interdisciplinary vascular conference comprising a vascular surgeon and an interventional radiologist. Cases were discussed based on the patient’s history, clinical symptoms, comorbidities, the possible treatment success and CTA findings in compliance with the TASC guidelines [1]. In case of emergency, the run-off CTA study was presented to and discussed with the vascular surgeon immediately after image acquisition by the interventional radiologist. In the conference, diagnostic confidence (DC) of run-off CTA was evaluated using a dichotomous scale and the intended therapy was documented. If run-off CTA provided adequate information regarding the vascular status of the patient to derive a treatment decision, diagnostic confidence was rated sufficient (DC+). If further imaging was requested by at least one of the two physicians in order to derive a treatment decision, diagnostic confidence of run-off CTA was rated insufficient (DC−).

### Image quality

Image quality (IQ) was evaluated retrospectively in consensus by two radiologists (4 and 9 years of clinical experience in interpreting run-off CTA), who were blinded to the patient’s clinical history and symptoms. IQ was assessed using MIP, CPR and axial datasets at four separate levels comprising the abdominopelvic level (suprarenal aorta—external iliac arteries), the thigh level (common femoral arteries—P3 segment of the popliteal arteries) the calf level (tibiofibular trunk—supramalleolar calf arteries) and the foot level (juxtamalleolar calf arteries—distal foot arteries). If vessel opacification at a respective level in comparison to the adjacent soft tissue allowed adequate visualization of margins, plaques, aneurysms, thrombi or occlusions, the level was rated as diagnostic (IQ+). In case of insufficient luminal enhancement (<100 HU) or vessel wall calcifications compromising assessment of the lumen in at least one major artery, the level was rated as non-diagnostic (IQ-).

### Reliability of the treatment decision

To assess the reliability of the therapeutic decision based on run-off CTA as documented by the interventional vascular conference, the therapy undertaken with possible modifications within 2 days after revascularisation was compared with the intended therapy in terms of agreement or disagreement in all patients with sufficient diagnostic confidence of run-off CTA (DC+). The mean time interval between run-off CTA and revascularisation therapy was assessed.

### Statistical analysis

Statistical analysis was conducted using SPSS software (IBM SPSS Statistics version 20; Chicago, Illinois).

Mean age, body mass index (BMI) as well as the standard deviation were calculated for several subgroups (patient sex, clinical manifestation of PAD and overall diagnostic confidence). After pre-testing for a Gaussian distribution using the Kolmogorov-Smirnov test, a t-test was applied to compare age and BMI in female and male patients depending firstly, on the clinical manifestation of PAD (acute vs. chronic) and secondly, on the diagnostic confidence (DC+ vs. DC−).

A χ^2^-test was applied to investigate a potential association between the presence of cardiovascular risk factors (arterial hypertension, nicotine abuse, hyperlipidemia, diabetes mellitus, impaired renal function, chronic haemodialysis) and either the clinical manifestation of PAD or the diagnostic confidence (DC) of run-off CTA.

To investigate a potential difference of the frequency of severe PAD between patients with acute and chronic PAD, a χ^2^-test was used.

To compare the frequency of arterial levels with non-diagnostic image quality dependent on clinical manifestation of PAD, arterial level and cause for non-diagnostic image quality, a χ^2^-test was applied.

A potential difference between the reliability of the treatment decision between patients with acute and chronic PAD was compared in patients undergoing revascularisation therapy using the χ^2^-test. For all statistical calculations a P-value <0.05 was considered significant.

## Results

The clinical manifestation of PAD was acute in 93 patients and chronic in 221 patients (Fig. 1). Mean age of patients with acute or chronic PAD showed no significant difference in men (Table 1), whereas women with acute PAD were significantly older than women with chronic manifestation (mean age 79 vs. 69 years; p < 0.001). As expected, there were significantly more patients with moderate PAD manifestation (Fontaine Stage IIa and IIb) in the subset of patients with chronic manifestation (P <0.001) and more patients with critical limb ischemia (Fontaine Stage III and IV) in the subgroup of patients with acute PAD (P < 0.001, Table 2).

**Table 1:** Demographics of patients with acute or chronic PAD depending on run-off CTA with sufficient (DC+) and insufficient diagnostic confidence (DC−).

	**Clinical manifestation of PAD**			
	***Diagnostic confidence***			
**Characteristic**	**acute PAD**	**chronic PAD**		**P-Value**
	***DC +***	***DC +***	***DC −***	
**Overall**				
***Number of Patients***	**93**	**221**		
		***215***	***6***	
**Female**				
***Number of* p*atients***	48	62		
		61	1	
***Mean Age [a]***	79 ± 11	69 ±11		**<0.001**
		69 ±11	62	
***Mean BMI [kg/m*** ^*2*^ ***]***	25.3 ± 4.3	25.3 ± 5.5		0.99
		25.3 ± 5.5	26.6	
**Male**				
***Number of Patients***	45	159		
		154	5	
***Mean Age [a]***	67 ± 11	67 ± 9		0.87
		67 ± 9	72 ± 10	0.20
***Mean BMI [kg/m*** ^*2*^ ***]***	26.2 ± 4.9	26.6 ± 4.7		0.57
		26.6 ± 4.6	27.4 ± 6.0	0.71

DC+, diagnostic confident run-off CTA.

DC−, non-diagnostic run-off CTA.

mean value ± standard deviation.

P-value of T-test.

**Table 2:** Fontaine Stage of patients with acute or chronic PAD depending on run-off CTA with sufficient (DC+) and insufficient diagnostic confidence (DC−).

	**clinical manifestation of PAD**			
	***diagnostic confidence (DC)***			
**fontaine stage**	**acute PAD**	**chronic PAD**		**P-Value**
	*DC+*	*DC+*	*DC−*	
***mild/moderate PAD***	9% (8/93)	55% (121/221)		**< 0.001**
- Stage IIa	0	27		
		27	0	
- Stage IIb	8	94		
		92	2	
***severe PAD***	91% (85/93)	45% (100/221)		**< 0.001**
- Stage III	72	40		
		39	2	
- Stage IV	13	59		
		57	2	

P-Value of χ^2^-test.

### Diagnostic confidence

In all patients presenting with acute PAD (Fig. 1), diagnostic confidence of run-off CTA was sufficient (DC+). In patients presenting with chronic PAD, diagnostic confidence of run-off CTA was sufficient to derive a treatment decision in 97% of the patients. In six patients (Fontaine Stage IIb: 2; III: 2; IV: 2), diagnostic confidence of run-off CTA was rated insufficient (DC−) and further imaging (DSA) was requested. In all six patients with insufficient diagnostic confidence of run-off CTA, IQ of the calf and foot level was non-diagnostic (IQ-) either due to insufficient vessel enhancement (2 patients) or due to severe vessel calcifications (4 patients). In these patients the visualisation of the calf arteries was important as no relevant stenoses accountable for the clinical symptoms were found in the assessable proximal part of the run-off CTA in 5 of 6 patients. In one patient, adequate visualisation of the calf arteries was required for femorocrural bypass planning.

With regard to the cardiovascular risk factors, nicotine abuse (73% vs. 60%, P = 0.02), hyperlipidaemia (56% vs. 42%, P = 0.03) and chronic haemodialysis (9% vs. 2%; P = 0.03) were significantly more frequently present in patients with chronic PAD as compared to those with acute PAD (Table 3).

**Table 3:** Presence of cardiovascular risk factors in patients with acute or chronic PAD depending on run-off CTA with sufficient (DC+) and insufficient diagnostic confidence (DC−).

	**clinical manifestation of PAD**			
	***diagnostic confidence (DC)***			
**cardiovascular risk factor**	**acute PAD**	**chronic PAD**		**P-Value**
	***DC +***	***DC +***	***DC −***	
**arterial hypertension**	83% (77)	81% (180)		0.77
		81% (174)	100% (6)	0.24
**nicotine abuse**	60% (56)	73% (162)		**0.02**
		73% (158)	67% (4)	0.71
**hyperlipidaemia**	42% (39)	56% (123)		**0.03**
		55% (118)	83% (5)	0.06
**diabetes mellitus**	34% (32)	35% (79)		0.82
		35% (75)	67% (4)	0.11
**impaired renal function**	39% (36)	31% (69)		0.20
		31% (66)	67% (4)	0.05
**chronic haemodialysis**	2% (2)	9% (20)		**0.03**
		8% (17)	50% (3)	**<0.001**

Note: In all patients with acute PAD, diagnostic confidence of run-off CTA was sufficient (DC+). P-Values of the χ^2^-test. The absolute numbers of patients are given in parenthesis.

In the subset of patients with chronic PAD, the cardiovascular risk factor chronic haemodialysis was significantly more frequent in the subgroup of patients with insufficient diagnostic confident run-off CTA as compared to the subgroup of patients with sufficient diagnostic confident run-off CTA (chronic haemodialysis, DC−: 50%, DC+: 8%, P < 0.001). There were no significant differences in the frequency of arterial hypertension, hyperlipidemia, nicotine abuse, diabetes mellitus and impaired renal function among the subgroups of patients with sufficient and insufficient diagnostic confident run-off CTA.

### Image quality

At the abdominopelvic level, image quality was non-diagnostic in none of the patients regardless of the clinical manifestation of PAD. At the thigh artery level, IQ was non-diagnostic in 0% of the patients with chronic PAD, and in 6% (6/93) of the patients with acute PAD. In patients with non-diagnostic IQ arteries of at least one side were unenhanced due to an acute occlusion.

**Fig 2:**
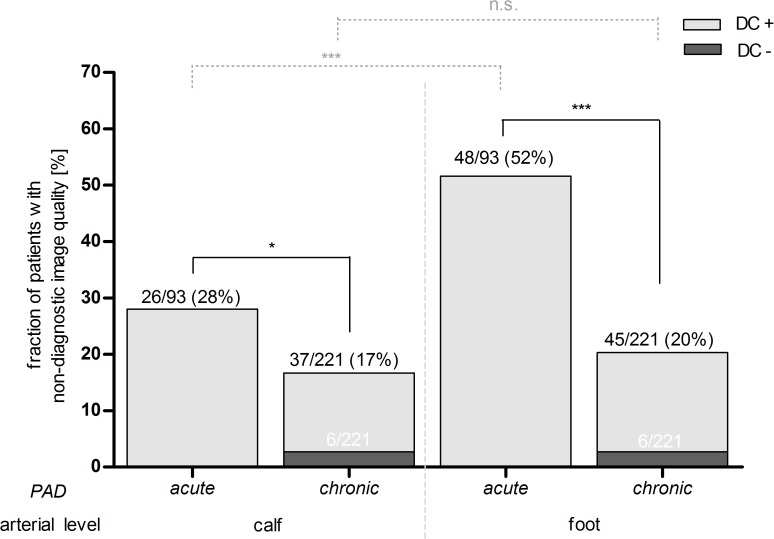
Proportion of patients with non-diagnostic image quality (IQ-) dependent on peripheral arterial disease (PAD) manifestation and arterial level. The total number of patients (black numbers) as well as the subset of patients with insufficient diagnostic confidence (DC−, white numbers) is given for each subgroup. DC− = insufficient, DC+ = sufficient diagnostic confidence of run-off CTA. * = P<0.05, *** = P<0.001, n.s. = no significance, P-Value of χ^2^-test.

At the calf level (Fig. 2), the portion of patients with non-diagnostic IQ increased and was significantly higher in patients presenting with acute PAD as compared to patients presenting with chronic PAD (acute: 28%; chronic: 17%, P = 0.02).

At the foot level, IQ deteriorated significantly as compared to the calf artery level in patients with acute PAD (foot level: 52%, P < 0.001), but not in patients with chronic PAD (foot level: 20%, P = 0.32).

**Fig 3:**
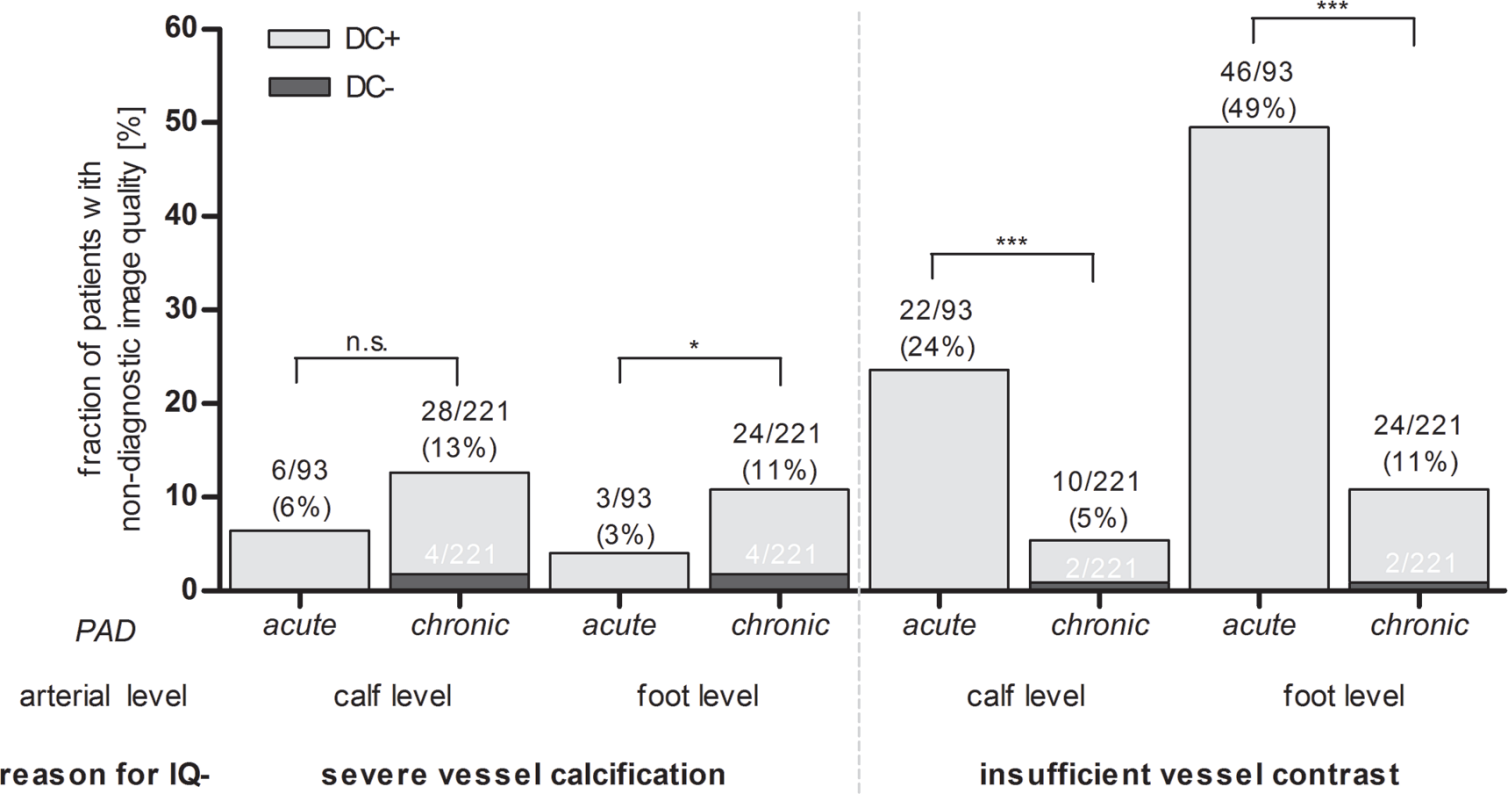
Proportion of patients with non-diagnostic image quality (IQ-) due to severe vessel calcifications (A) or insufficient vessel contrast (B) dependent on peripheral arterial disease (PAD) manifestation and arterial level. The total number of patients (black numbers) as well as the subset of patients with insufficient diagnostic confidence of run-off CTA (DC−, white numbers) is given for each subgroup. DC− = insufficient, DC+ = sufficient diagnostic confidence. * = P <0.05, *** = P<0.001, n.s. = no significance, P-Value of χ^2^-test.

With regards to artery levels with non-diagnostic IQ (Fig. 3), insufficient vessel enhancement was significantly more frequently observed at the calf (acute: 24% vs. chronic: 5%, P<0.001) and foot level (acute: 49% vs. chronic: 11%, P<0.001) in patients with acute PAD as compared to patients with chronic PAD. Severe vessel wall calcifications were not significantly more frequently observed at the calf level compared between patients with acute and chronic PAD. At the foot level severe calcifications were significantly more often the reason for insufficient vessel delineation (acute: 3% vs. chronic: 11%, P = 0.03) in patients with chronic PAD compared to patients with acute PAD. The risk that the diagnostic confidence of a run-off CTA in patients with chronic PAD was insufficient, if the image quality was non-diagnostic, was approximately 16% (6/37).

### Reliability of the treatment decision

The mean time and [90% percentile] between run-off CTA and invasive revascularisation therapy in patients with acute PAD was 2 d [5 d] and for patients with chronic PAD 20 d [54 d]. Agreement of intended and undertaken surgical or endovascular treatment was significantly more frequently observed in patients with chronic PAD (123/133 = 92%) as compared to patients with acute PAD (64/78 = 82%, P = 0.02) as demonstrated in Tables 4 and 5.

**Table 4:** Intended and undertaken therapy in patients with *acute PAD*.

**intended therapy**			**number of patients**				**undertaken therapy** *
			**Σ**	**refused**	**agree-ment**	**disagree-ment** *	
**surgical treatment**			**70/93**	**2/70**	**56/68**	**12/68**	
			(75%)	(3%)	(81%)	(19%)	
Thrombectomy			48	1	35	4	major amputation
						5	additional endovascular catheter-directed thrombolysis
						3	additional PTA ± Stenting
bypass surgery							
		*aortoiliac / aortofemoral*	10		10		
		*femoropopliteal*	4		4		
		*femorocrural*	3		3		
Amputation							
		*major*	4	1	3		
		*minor*	1		1		
**endovascular treatment**			**12/93**	**2/12**	**8/10**	**2/10**	
			(13%)	(17%)	(80%)	(20%)	
PTA ± Stenting							
	*pelvic arteries*		4		4		
	*thigh arteries*		3	1	2		
	*- calf arteries*		2	1	1		
thrombectomy			2		1	1	conservative; endovascular bypass recanalization failed
thrombolysis			1			1	conservative; Catecholamine induced circulatory disorder
**non invasive treatment**			**11/93**				
			(12%)				
no therapeutic option for endovascular or surgical revascularization			6				
catecholamine induced circulatory disorder			1				
spontaneous improvement			1				
inoperable due to comorbidities			3				

*The conducted therapy is given for all patients planned to undergo endovascular or surgical treatment in case of disagreement. Note that diagnostic confidence of all run-off CTA studies in patients with acute PAD was rated to be sufficient (DC+). PTA = percutaneous transluminal angioplasty.

**Table 5:** Intended and undertaken therapy in patients with chronic PAD and diagnostic confidence of run-off CTA rated to be sufficient (DC+).

**intended therapy**			**number of patients**				**undertaken therapy** *
			**Σ**	**refused**	**agree-ment**	**disagree-ment** *	
**surgical treatment**			**79/215**	**6/79**	**66/73**	**7/73**	
			(37%)	(9%)	(90%)	(10%)	
thrombectomy/ endarterectomy			18		12	5	additional PTA ± Stenting
						1	additional Bypass
bypass surgery							
	*aortoiliac / aortofemoral*		19	4	*14*	1	thrombus aspiration, bypass extension
	*femoro-popliteal*		22	2	20		
	*femorocrural*		6		6		
amputation							
		*major*	7		7		
		*minor*	7		7		
**endovascular treatment**			**68/215**	**8/68**	**57/60**	**3/60**	
			(32%)	(12%)	(95%)	(5%)	
PTA ± Stenting							
	*pelvic arteries*		34	3	28	3	no indication for PTA
	*thigh arteries*		29	5	24		
	*limb arteries*		5		5		
**non-invasive treatment**			**68/215**				
			(31%)				
no relevant stenosis			21				
no indication for revascularization.			28				
no therapeutic option for endovascular or surgical revascularization			11				
inoperable due to comorbidities			8				

*The undertaken therapy is given for all patients planned to undergo endovascular or surgical treatment in case of disagreement. PTA = percutaneous transluminal angioplasty.

**Fig 4:**
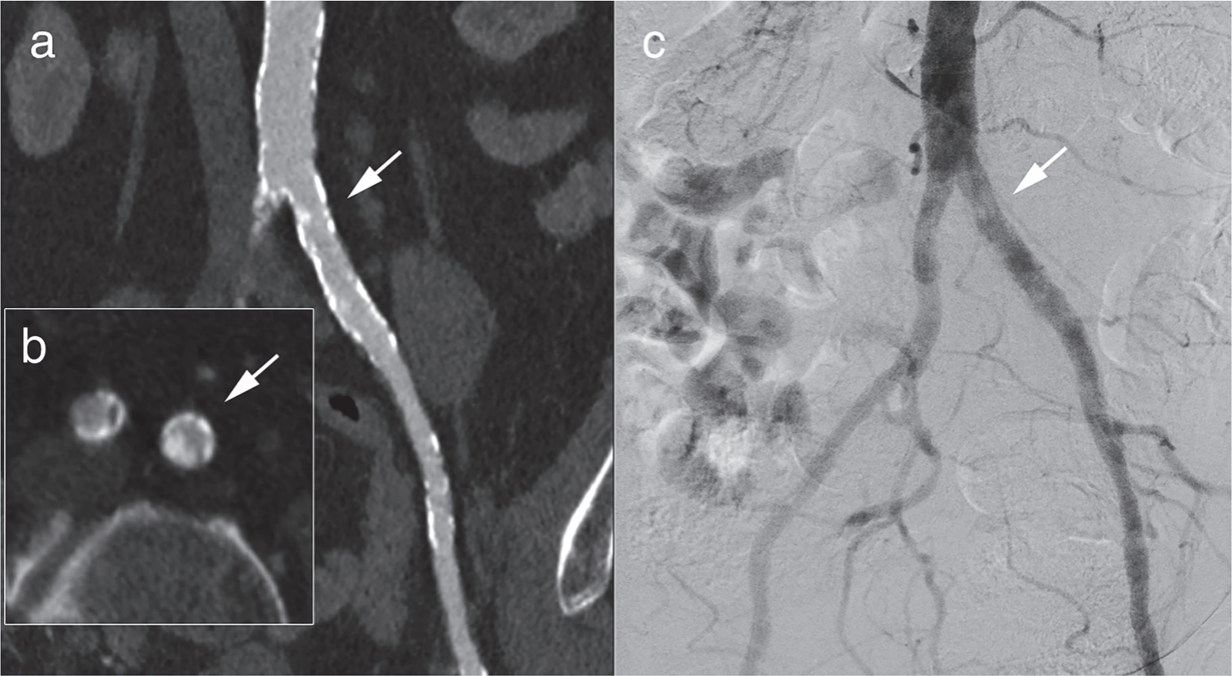
Example of a run-off CTA with sufficient diagnostic confidence and diagnostic image quality and disagreement of intended and undertaken therapy. 69 y old male presenting with Fontaine stage IIb of the left leg. Run-off CTA revealed a calcified stenosis rated to be haemodynamic relevant in the curved multiplanar reformat (a) and the axial image (b, white arrow). In DSA (c), the stenosis could not be confirmed.

This was mainly attributable to a change of the therapeutic concept during the surgical or endovascular procedure as given in detail in Table 6. The most frequent reason for a modification of the revascularisation therapy in patients with chronic PAD was a prior hidden stenosis after thrombectomy. In patients with acute PAD the main reason for a modification of the revascularisation therapy was a new thrombosis requiring catheter derived thrombolysis or amputation. In four patients (acute PAD: 1 patient, chronic PAD: 3 patients), false-positive findings of run-off CTA were accountable for disagreement (Fig. 4).

**Table 6:** Reasons for disagreement between intended and undertaken therapy.

**PAD**	**Reason**	**number of patients**
**chronic**		
	hidden stenoses	4
	overestimation of stenoses	3
	new thrombosis	1
	failed thrombectomy	1
	arterial dissection	1
**Acute**		
	new thrombosis	9
	hidden stenoses	2
	arterial Dissection	1
	overestimation of stenoses	1
	failed recanalisation	1

## Discussion

The results of this study demonstrate that the rate of run-off CTA with sufficient diagnostic confidence in clinical routine is very high and the treatment decision in patients undergoing revascularisation therapy is reliable although the image quality of the calf and foot arteries is considerable more often non-diagnostic compared to the rate of run-off CTA with sufficient diagnostic confidence. This implies that a perfect visualisation of the calf and foot arteries may not always be required for the treatment decision. This is important as most previous published studies focused on the sensitivity and specificity of stenosis quantification only and did not take into account that treatment decision requires primarily a comprehensive assessment of the vascular territory. Accurate quantification of each single stenosis in an artery with numerous stenoses becomes less important for the treatment decision than the extent of vessel wall calcifications or the relation of plaques to vessel bifurcations with regard to the decision whether an endovascular or surgical approach should be chosen. [1,2,4].

**Fig 5:**
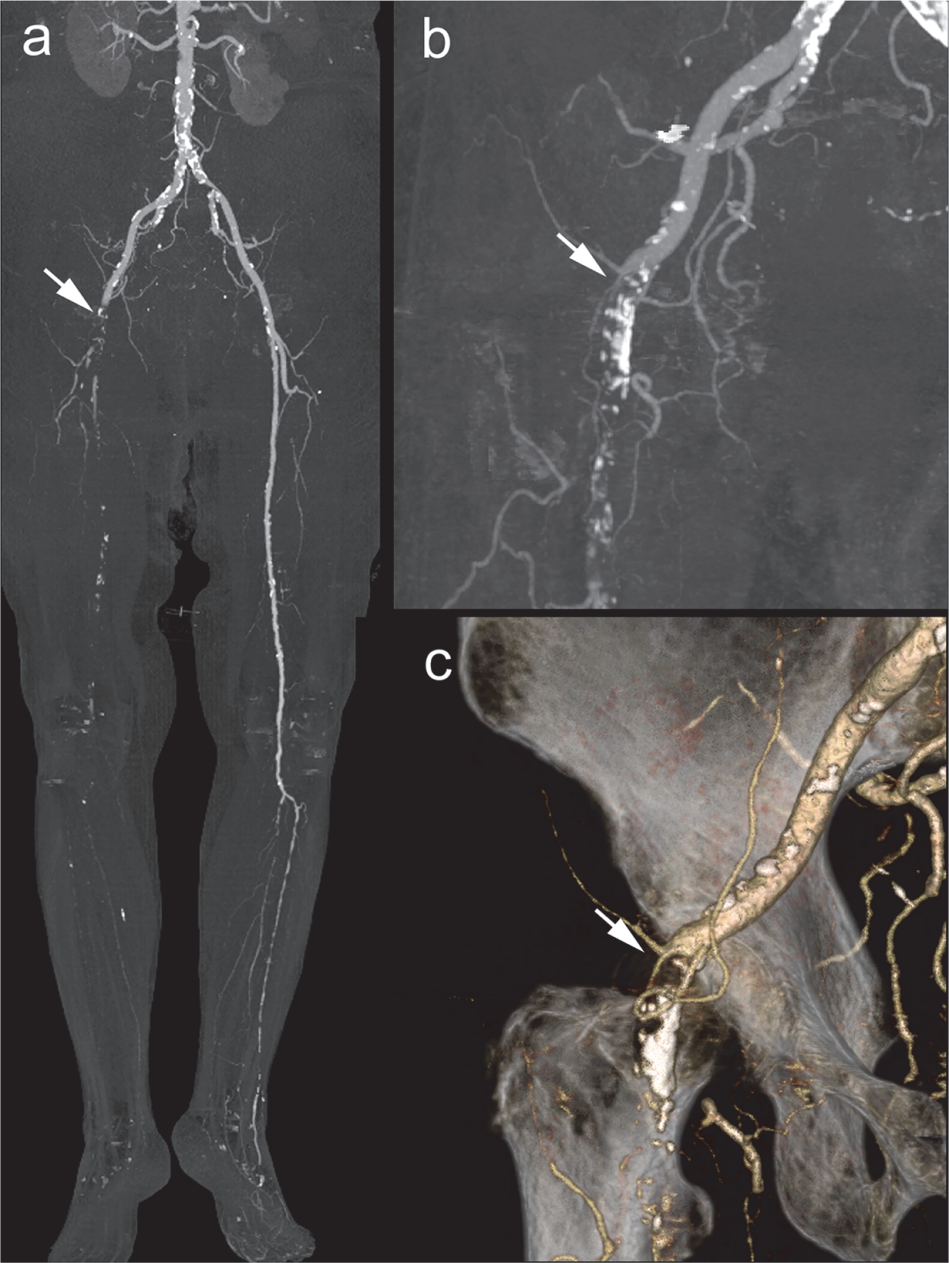
Example of a run-off CTA with sufficient diagnostic confidence despite non-diagnostic image quality. 84 y old female with acute onset of severe right leg pain. MIP (a,b) and volume rendering technique image (c) of run-off CTA revealed a thromboembolic occlusion with lack of arterial enhancement of the large arteries of the right leg from the external iliac artery (white arrow) down to the foot. Immediate surgical thrombectomy confirmed the finding of run-off CTA.

In patients with acute PAD, diagnostic confidence was sufficient in all run-off CTA studies while a non-diagnostic image quality was present in 28% of the calf and 52% of the foot arteries. The non-diagnostic image quality observed in our study was predominantly attributed to an insufficient vessel enhancement caused by an occlusion and missing inflow of contrast-enhanced blood (Fig. 5). Considering the acute onset and manifestation of the clinical symptoms, lack of arterial enhancement did not impair the therapeutic decision as it furthermore represented a key imaging finding of an acute occlusion.

**Fig 6:**
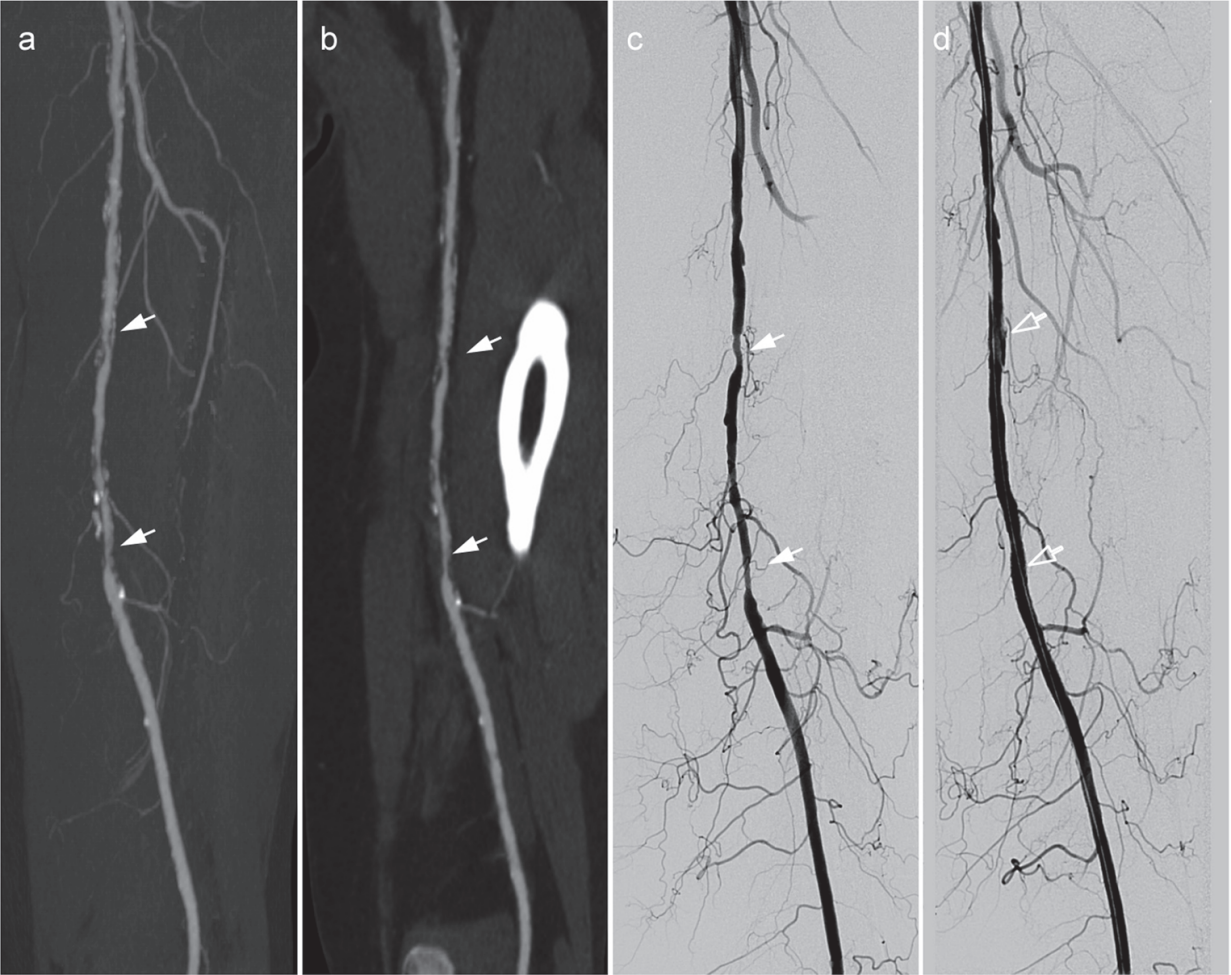
Example of a run-off CTA with sufficient diagnostic confidence and diagnostic image quality. 69 y old female with intermittent claudication of the left lower leg (Fontaine stage IIB). Run-off CTA showed multiple stenoses (white arrows) of the left superficial femoral artery (TASC B) in the MIP images (a) and curved MPR (b). Stenoses were comfirmed by DSA (c) and successfully treated by percutaneous transluminal angioplasty and stenting (d, empty white arrow).

In patients with chronic PAD, diagnostic confidence was rated sufficient in 97% of run-off CTA studies (Fig. 6); non-diagnostic image quality was predominantly caused by severe vessel calcifications in 13% of the calf arteries while in the foot level no significant difference between the frequency of calcifications and insufficient arterial enhancement was observed.

This portion of run-off CTA studies with sufficient diagnostic confidence and non-diagnostic image quality was found in patients with relevant stenoses or occlusions in the proximal regions of run-off CTA with diagnostic image quality. With respect to the TASC-guidelines, the additional information about the non-diagnostic regions of calf or foot arteries would not have altered the treatment approach in this patient group [1].

In all patients with insufficient diagnostic confidence of run-off CTA, only the image quality of the calf and foot arteries was non-diagnostic. In most of these patients, the abdominopelvic and thigh arteries revealed no significant stenoses and therefore a further evaluation with DSA was mandatory.

The high reliability of treatment decisions based on run-off CTA with sufficient diagnostic confidence was shown by the high agreement of intended and undertaken therapy in patients undergoing revascularization. The higher rate of disagreement of the intended and undertaken therapy in patients with acute PAD can be attributed to intraoperative complications, additional interventional procedures to treat previously hidden stenoses in occluded arteries or technical failure of surgical procedures. However, DSA imaging would neither alter the depiction of an occluded vessel nor reduce the intraoperative complication rate. Overall, the observed failure rate of surgical treatment was comparable to that reported in the literature [14].

Run-off CTA is known as one of the most challenging examinations in computed tomography with a long cranio-caudal coverage and a relative high risk of non-diagnostic image quality of the calf and foot arteries due to an insufficient arterial opacification, venous contamination or compromising vessel calcifications [15]. These drawbacks are mainly caused by the static characteristics of run-off CTA as compared to dynamic examination such as DSA or MRA [16,17]. Further disadvantages of the run-off CTA are the need for radiation exposure to the patient and the application of potentially nephrotoxic iodinated contrast material. This is important as patients with PAD often suffer from accompanying renal insufficiency. Therefore the indication for run-off CTA in patients with an advanced kidney disease and correspondingly high risk of contrast induced nephropathy (CIN) should be carefully made and other alternatives e.g. carbon dioxide angiography should be considered [18,19].

Radiation exposure of a run-off CTA is usually of minor importance as compared to the risk of CIN due to a limited life expectancy in this specific patient cohort [1]. The radiation exposure of a run-off CTA is normally lower compared to a standard abdominal CT due to depiction of high contrast objects with therefore higher tolerable image noise and lower possible tube current settings [2,20,21].

In this study we used a robust simple and time-efficient contrast material application protocol combined with a constant preselected table feed to achieve an adequate arterial opacification and a simple patient selection to minimize the risk of compromising arterial vessel calcifications [15].

For patients with chronic PAD, the observed image quality was moderately lower as compared to the published results. Siriapisith et al. [22] observed adequate arterial opacification in 97% of the calf arteries and in 11% of the foot segments. Meyer et al. [15] obtained diagnostic visualization of the foot arteries in 90% of patients with chronic PAD. The differences might be attributed to a higher percentage of patients with severe PAD, diabetes mellitus and impaired renal function leading to a higher risk of severe vessel calcifications. If only the arterial opacification is considered, the results are similar, as Siriapisith et al. excluded patients with severe vessel calcifications. For patients with acute PAD, this is the first study demonstrating the high diagnostic confidence of run-off CTA. As suggested in the literature, a more robust arterial enhancement of the calf and foot arteries might be achieved using test bolus techniques, individual table feeds or to acquire a second dataset [23] or to use time resolved imaging techniques [11,15,24,25]. This strategy causes additional radiation exposure, which is indeed comparably low at the lower limb due to a very small conversion factor and low-tube currents if tube current modulation techniques are applied [26,27].

Another option to increase the proportion of diagnostic images would be to apply more restrictive patient selection criteria to decrease the number of patients with chronic diabetes and haemodialysis [7]. However, the low number of run-off CTA with insufficient diagnostic confidence implies that a more sophisticated and potentially more time consuming acquisition protocol or a more restrictive patient selection are not necessary. This underlines the clinical value of run-off CTA as a cost-efficient imaging modality compared to DSA or MRA [28]. However the continuous development of the CT techniques with more efficient X-ray tubes, detector systems and the introduction of new reconstruction algorithms bears the potential to substantially decrease the radiation burden of a run-off CTA and to minimize the needed iodinated contrast material [29]. Furthermore the improvement of the PACS—systems handling the huge data amounts as well as the introduction of thin-client systems for image interpretation and presentations in the recent years might enable a further simplification of the implementation of run-off CTA in clinical practice.

Our study has certain limitations. First, based on its retrospective design, a long-term follow up of patient outcome after treatment was not assessed. Instead, intraoperative findings of patients undergoing surgical revascularization, technical success of the surgical procedure and short term follow-up after the procedure until the patient was discharged served as reference standard. In patients treated conservatively, no confirmation with regards to the treatment decision was available. However, during the study period no surgical or endovascular revascularisation was undertaken in these patients in our hospital. Second the decision of the diagnostic confidence was not blinded, as cases were discussed based on the patient’s history, clinical symptoms, comorbidities and the possible treatment success. Third the image quality of run-off CTA was assessed by two readers in consensus, therefore interobserver variability could not be calculated. Finally there were only a few cases of run-off CTA with insufficient diagnostic confidence, which on the one hand might limit the statistics but on the other hand demonstrates the reliability of the run-off CTA.

## Conclusion

Run-off CTA is a reliable imaging modality for primary diagnostic work-up of patients with acute and chronic PAD.
